# Prevalence and Characterization of Coagulase Positive *Staphylococci* from Food Products and Human Specimens in Egypt

**DOI:** 10.3390/antibiotics10010075

**Published:** 2021-01-14

**Authors:** Eman E. Abdeen, Walid S. Mousa, Sarah Y. Abdelsalam, Hanim S. Heikal, Reyad R. Shawish, Mohammed Nooruzzaman, Mohamed M. Soliman, Gaber E. Batiha, Ahmed Hamad, Ahmed Abdeen

**Affiliations:** 1 Department of Bacteriology, Mycology and Immunology, Faculty of Veterinary Medicine, University of Sadat City, Sadat City 32897, Egypt; 2 Department of Animal Medicine and Infectious Diseases, Faculty of Veterinary Medicine, University of Sadat City, Sadat City 32897, Egypt; walid.saad@vet.usc.edu.eg; 3 Division of Public Health, Veterinary Hospital, Khanka, Benha 13736, Egypt; sarah.yosri@vet.usc.edu.eg; 4 Department of Husbandry and Animal Wealth Development, Faculty of Veterinary Medicine, University of Sadat City, Sadat City 32897, Egypt; hanem.hekal@vet.usc.edu.eg; 5 Department of Food Hygiene and Control, Faculty of Veterinary Medicine, University of Sadat City, Sadat City 32897, Egypt; reyad.rabea@vet.usc.edu.eg; 6 Department of Pathology, Faculty of Veterinary Science, Bangladesh Agricultural University, Mymensingh 2202, Bangladesh; mohammed.nooruzzaman@bau.edu.bd; 7 Clinical Laboratory Sciences Department, Turabah University College, Taif University, Taif 21995, Saudi Arabia; mmsoliman@tu.edu.sa; 8 Department of Biochemistry, Faculty of Veterinary Medicine, Benha University, Toukh 13736, Egypt; 9 Department of Pharmacology and Therapeutics, Faculty of Veterinary Medicine, Damanhour University, Damanhour 22511, Egypt; dr_gaber_batiha@vetmed.dmu.edu.eg; 10 Center of Excellence for Screening of Environmental Contaminants, Benha University, Toukh 13736, Egypt; ahmed.alhussaini@fvtm.bu.edu.eg (A.H.); ahmed.abdeen@fvtm.bu.edu.eg (A.A.); 11 Department of Food Hygiene and Control, Faculty of Veterinary Medicine, Benha University, Toukh 13736, Egypt; 12 Department of Forensic Medicine and Toxicology, Faculty of Veterinary Medicine, Benha University, Toukh 13736, Egypt

**Keywords:** *Staphylococcus aureus*, antimicrobial resistance, food safety, food products, *mecA* gene

## Abstract

Methicillin-resistant *Staphylococcus aureus* (MRSA) strains have veterinary and public health importance as they are responsible for a wide range of difficult to treat infections and food poisoning. Two hundred samples (50 samples each of minced meat, beef luncheon, Karish cheese, and human samples (pus swab from open wounds)) were cultured, and MRSA strains were identified using disk diffusion tests and *mecA* gene-based PCR. A total of 35% (70/200) of the examined samples were confirmed as coagulase-positive *S. aureus* in minced meat (46%), beef luncheon (44%), Karish cheese (44%), and human samples (22%). The MRSA strains showed resistance to amoxicillin (91.4%), penicillin (97.1%), cefoxitin (85.7%), cephradine (82.9%), tetracycline (57.2%), and erythromycin (52.8%). More than half of the tested *S. aureus* isolates harbored the *mecA* gene. The sequence analysis of the *mecA* gene from the minced meat, Karish cheese, and human samples revealed high genetic similarities between the *S. aureus* isolates from these sources. In conclusion, our findings indicate a risk for the transmission of the *mecA* gene of *S. aureus* across the food chain between humans and animal food products. Further studies should focus on finding additional epidemiological aspects of the MRSA strains in food chain.

## 1. Introduction

*Staphylococcus aureus* is recognized as a zoonotic pathogen of public health concern, producing serious illness in humans, subclinical mastitis in cows, and outbreaks of food poisoning [1,2]. Globally, *S. aureus* causes widely distributed opportunistic, nosocomial, and population-related diseases [3]. A recent report stated that *S. aureus* strains can spread to different geographical regions through food and milk products [4]. According to a surveillance study in India, a lack of hygienic procedures during processing and post-processing handling of food products, particularly those of animal origins such as eggs, meat, and milk, facilitated microbial contamination [5]. 

*S. aureus* has developed several virulence factors that aid in pathogenesis and udder infection, such as evasion and adhesion within the host and resistance to the immunologic responses [6,7]. 

Production of coagulase is a crucial phenotypic feature and the major determinant factor for identifying *S. aureus* strains. Moreover, the variability at the 3′ end coding region of the *coa* gene has been used for genotyping of *S. aureus* strains from humans and animals [8].

Methicillin-resistant *S. aureus* (MRSA) has frequently been isolated from livestock, and the possibility of zoonotic spread to humans has been recognized. For example, the MRSA ST398, a model clone of livestock-associated MRSA (LA-MRSA), has been found in human population that are in frequent contact with animals such as farmers or veterinarians [9]. Outbreaks of the MRSA infection have been reported in the USA, Europe, and Egypt [10,11]. 

Several studies have highlighted the evolution of resistance to numerous antimicrobial drugs in *S. aureus* strains obtained from bovines [12]. *S. aureus* has also been implicated in food poisoning outbreaks, healthcare, and community-associated infection, particularly MRSA strains that exhibit multidrug resistance (MDR) [11,13,14]. The widespread predominance of MDR-MRSA strains poses a significant risk to public health, and invasion of MDR strains could theoretically transfer resistance genes to humans [13].

In fact, certain bacterial strains of the genus *Staphylococcus* that have developed resistance to various antibiotics carry the *mecA* gene that encodes penicillin-binding protein 2a (PBP2a) and facilitate resistance to methicillin and other β-lactam antibiotics, rendering them a global public health issue [14]. Molecular identification using PCR is the gold standard approach for identifying the *mecA* gene between MRSA strains [15]. However, a special *mecA* homolog gene was detected in some *S. aureus* strains, primarily the mecALGA251 (*mecC*), and this divergent *mecA* gene counterpart with 70% homology to the *mecA* gene has been designated as *mecC* [16]. 

Karish cheese is a common local and popular type of white, soft, lactic cheese made from raw milk or laban rayeb (raw cottage cheese with salt) which is not exposed to any heat treatment. Additionally, minced meat and beef luncheon are very common and popular raw meat products consumed by the Egyptian people which require no or low heat treatment during cooking. The frequent consumption of such food products of animal origin in developing countries such as Egypt without health oversight can result in the spread of certain bacterial infections such as *S. aureus*. Therefore, this study aimed to investigate the prevalence and antibiogram profile of *S. aureus* isolates in animal food products such as minced meat, beef luncheon, and Karish cheese, as well as in human samples. Moreover, partial sequencing of the *mecA* gene of three MRSA strains has been performed to compare the identity of the *mecA* gene with related global strains as well as to highlight the possibility of transmitting the antibiotic resistance genes to humans through animal food products and its potential public health significance.

## 2. Materials and Methods 

### 2.1. Ethical Statement

The medical staff of Al-Qaluobyia Hospital collected the human samples used in this study following the rules and regulations of the Egyptian Ministry of Health under full medical supervision. Written consent has been obtained from the patients before using the specimens. Food samples (minced meet, beef luncheon, and Karish cheese) were collected randomly from local markets in the Al-Qaluobyia Governorate. 

### 2.2. Study Area

The study was conducted in the Al-Qaluobyia governorate in Lower Egypt, about 35.26 km north of Cairo in the Nile Delta region (30.41 N: _N 31.21_E at an altitude of 9.0 m). The study area (1124 km^2^) had a rapid urbanization over the last three decades. According to inhabitance estimates, in 2018, most residents in the governorate lived in rural areas, with an urbanization rate of only 44.7%. Out of 5,703,000 people located in the governorate, 2,509,000 people lived in rural areas and 3,194,000 lived in urban areas [17]. In addition, the Qaluobyia governorate is the leader in poultry and egg production and food packaging and processing in Egypt. 

### 2.3. Collection of Samples and Preparation

A total of 200 samples (50 samples each of minced meat, beef luncheon, Karish cheese, and human samples (pus swab from open wound)) were collected from December 2018 to December 2019. The food products samples were collected from different areas in the same governorate from shops (Al-Qalyubia governorate). Samples were collected under aseptic condition. The human pus samples were collected from hospitals in the same area by medical staff under the supervision of a physician. The samples were collected in accordance with Americana Public Health Association (APHA), 1992 [18] for the food samples and in accordance with Rice et al., 2016 for human samples [19]. The samples were transferred to the laboratory in sterile plastic bags under hygienic and aseptic cooled conditions for bacteriological examination. One gram of minced meat, beef luncheon, and Karish cheese was mashed with 9 mL of nutrient broth and incubated for 18 h at 37 °C as described previously [20]. The extracted pus swab from humans was grown in nutrient broth and incubated for 18 h at 37 °C under aseptic conditions in accordance with Rice et al. [19]. 

### 2.4. Phenotypic Isolation and Identification of Staphylococcus aureus

The processed samples were incubated for 1-2 days at 37 °C in Baird–Parker agar (Oxoid Ltd., Basingstoke, UK) and 10% sheep blood agar (Oxoid Ltd., UK). *Staphylococci* were confirmed through Gram staining, catalase, and coagulase test [20]. Virulence activities such as hemolytic activity and DNase agar testing were performed in accordance with Murray et al. [21]. Biofilm activity on Congo red medium was tested in accordance with Freeman et al. [22].

### 2.5. Antibiogram Profile of Staphylococcus aureus Isolates

The antibiogram profile of 70 *S. aureus* isolates was tested in vitro using the Kirby–Bauer disk diffusion method. A suspension of the bacteria was adjusted to a 0.5 McFarland suspension. Twelve antimicrobials disks (Oxoid Ltd., UK) of different antibiotic groups were used to detect the antimicrobial susceptibility and resistance patterns of these isolates. The following antibiotics were used: 100 IU penicillin (P), 25 µg amoxicillin (AX), 30 µg cefoxitin (FOX), 30 µg vancomycin (VA), 10 µg gentamicin (CN), 15 µg erythromycin (E), 30 µg tetracycline (TE), 5 µg ciprofloxacin (CIP), 10 µg norfloxacin (NOR), 30 µg cephradine (CE), chloramphenicol (C), and 1.25/23.75 µg trimethoprim-sulfamethoxazole (SXT). The results were interpreted as resistant, intermediate, or susceptible according to the inhibitory zone as described by the Clinical and Laboratory Standards Institute (CLSI) [23]. The *S. aureus* ATCC 25923 was used as a quality control organism in antimicrobial susceptibility determination.

### 2.6. Molecular Characterization of Staphylococcus aureus Isolates

DNA was extracted using the QIAamp DNA Mini Kit (Cat. No. 51304, Qiagen, Hilden, Germany). The list of primers used to amplify the *coa* and *mecA* genes of *S. aureus* isolates is provided in Table 1. For PCR, a reaction mixture of 50 µL volume was prepared, containing 25 µL of PCR Master Mix (Cat. No. 201445, Qiagen, Germany), 1 µL (10 pmol/µL) of each primer, 6 µL target DNA, and the remaining volume needed to reach 50 µL was adjusted with deionized water. The reaction was conducted in an Applied Biosystem 2720 thermal cycler (Applied Biosystems, Foster, CA, USA). The following thermal profile was used: Initial denaturation at 94 °C for 5 min; 40 cycles each consisting of denaturation at 94 °C for 30 sec, annealing at 55 °C (*coa* gene) or 57 °C (*mecA* gene) for 45 sec and extension at 72 °C for 30 sec; and finally extension at 72 °C for 5 min. After completing the run, PCR products (15 µL) were subjected to agarose gel electrophoresis (1.5%) and visualized under UV light in a gel documentation system.

**Table 1:** PCR primers and conditions for *coa* and *mec*A gene amplification.

Target	Primers	Sequences (5′ to 3′)	Amplicon Size (bp)	Annealing Temperature	References
*mecA*	Forward	AGA AGA TGG TAT GTG GAA GTT AG	583	57 °C	[24]
	Reverse	ATG TAT GTG CGA TTG TAT TGC			
*Coa*	Forward	ACC ACA AGG TAC TGA ATC AAC G	600–1000	55 °C	[25]
	Reverse	TGC TTT CGA TTG TTC GAT GC			

### 2.7. Sequencing and Analysis of Staphylococcus aureus mecA Gene

The PCR products were purified using GeneJET PCR Purification Kit (Cat no. K0701, ThermoFisher Scientific, USA) and sequenced from a commercial laboratory (GATC Biotech Company, Germany) in an ABI 3730xl DNA sequencer using both forward and reverse primers. To identify homologies in the nucleotide and amino acid sequences, the isolated strains were compared with *S. aureus* strains available in the GenBank using BLAST 2.0 and PSI- BLAST search databases (National Center for Biotechnology Information NCBI at http://www.ncbi.nlm.nih.gov/), respectively. The nucleotide sequence comparison and multiple alignments with reference strains and the deduced amino acid sequence analysis were conducted as described previously [26] using the BioEdit sequence alignment editor, CLUSTALX software for multiple sequence alignment. ClustalW software for multiple sequence alignment [27]. ClustalV [28] and MegAlign (DNASTAR, Lasergene^®^, Version 7.1.0, Madison, WI, USA) [29] Phylogenetic trees were constructed using MegAlign using the neighbor-joining method based on ClustalW. Bootstrapping values were estimated using a random seeding value of 111 [27]. ClustalV was applied when end gaps were faced. Sequence deviation and identity percent were calculated using MegAlign and the structural character of our protein sequence was identified using Protean (DNASTAR, Lasergene^®^, Version 7.1.0., Madison, WI, USA).

### 2.8. Statistical Analysis

To visualize the multidrug resistance profile of MRSA strains, an UpSetR plot was prepared using an online platform (https://gehlenborglab.shinyapps.io/upsetr/).

## 3. Results

### 3.1. Prevalence of Coagulase-positive Staphylococci Isolated from Food Products and Human Samples

At first, we investigated the prevalence of coagulase-positive *Staphylococci* (CPS) in animal food products and human specimens. To this end, we collected 200 samples of minced meat, beef luncheon, Karish cheese, and human pus swabs. Out of 200 samples, 123 samples showed phenotypically positive growth on Baird–Parker medium. The prevalence of CPS was 24 (48%), 42 (84%), 38 (76%), and 19 (38%) in minced meat, beef luncheon, Karish cheese, and human swabs samples, respectively (Table 2).

### 3.2. Biochemical Activity and Virulence Factors of the Coagulase-positive Staphylococci (CPS) Isolates

Next, we tested the biochemical and enzymatic activity of the CPS isolates. Out of 123 isolates, 70 strains were confirmed as coagulase-positive *S. aureus* by coagulase test. The hemolysis activity on blood agar media was found in 23 (46%), 22 (44%), 22 (44%), and 11 (22%) CPS isolates from minced meat, beef luncheon, Karish cheese, and human samples, respectively. In addition, the DNase activity was found in 24 (48%), 42 (84%), 38 (76%), and 19 (38%) CPS isolates detected in minced meat, beef luncheon, Karish cheese, and human samples, respectively. The lecithinase activity was noticed in 21 (42%), 2 (4%), and 1 (2%) CPS isolates from minced meat, beef luncheon, and human samples, respectively, while no isolates from Karish cheese exhibited lecithinase activity. Biofilm activity on Congo red medium was observed in 19 (38%), 37 (74%), 32 (64%), and 19 (38%) CPS isolates from minced meat, beef luncheon, Karish cheese, and human samples, respectively. Collectively, the CPS isolates detected in animal food products, and human samples carried potential virulence factors.

### 3.3. Antimicrobial Resistance of the Coagulase-positive Staphylococci (CPS) Isolates

We performed the antimicrobial resistance analysis of the purified *S. aureus* isolates obtained from animal food products and human samples using the disc diffusion technique. A total of 12 antibiotics belonging to eight antimicrobial classes were used in the analysis (Table 3). The antibiotic sensitivity test of the 70 strains of *S. aureus* revealed a relatively high antibiotic resistance property of these isolates. The *S. aureus* isolates showing resistance to antibiotics were as follows: penicillin (97.1%), amoxicillin (91.4%), cefoxitin (85.7%), cephradine (82.9%), tetracycline (57.2%), and erythromycin (52.8%). On the other hand, a relatively higher sensitivity of the *S. aureus* isolates was detected to vancomycin (84.3%), chloramphenicol (70%), norfloxacin (55.7%), trimethoprim-sulfamethoxazole (50%), gentamicin (44.3%), and ciprofloxacin (42.8%) (Table 3). Collectively, the antimicrobial sensitivity assay revealed the presence of a large number of antibiotic-resistant *S. aureus* isolates from animal food products.

### 3.4. Multidrug Resistance (MDR) Profiles of Methicillin-Resistant Staphylococcus aureus (MRSA) Strains from Animal Food Products and Human Samples

**Figure 1:**
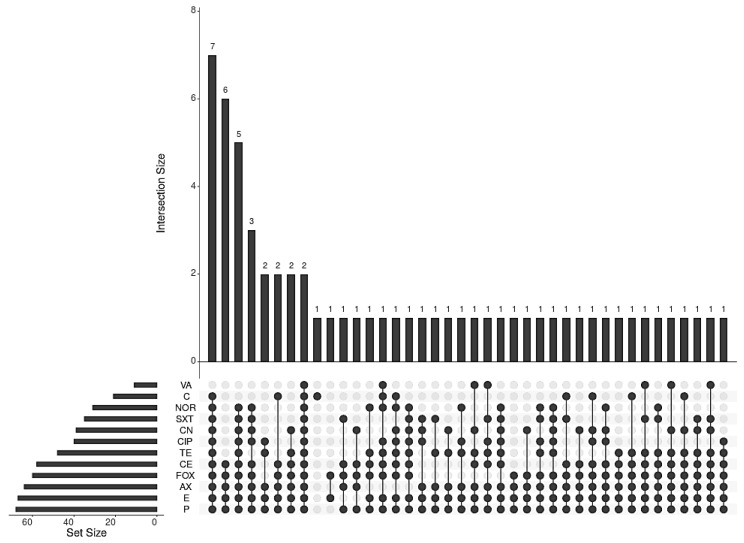
An UpSetR plot is showing multidrug-resistant profile among the 70 *Staphylococcus aureus* strains detected in animal food products and human pus samples. Note: penicillin (P), amoxicillin (AX), cefoxitin (FOX), vancomycin (VA), gentamicin (CN), erythromycin (E), tetracycline (TE), ciprofloxacin (CIP), norfloxacin (NOR), trimethoprim-sulfamethoxazole (SXT), chloramphenicol (C), cephradine (CE).

The multidrug resistance (MDR) profiles of the 70 *S. aureus* isolates were analyzed using 12 different antibiotics belonging to eight antimicrobial classes. The MDR profiles of the *S. aureus* isolates are presented in Figure 1 and Supplementary Table S1. Of note, two of the *S. aureus* isolates showed resistance to all 12 antibiotics belonging to eight antimicrobial classes. Seven isolates showed resistance of 11 antibiotics belonging to seven antimicrobial classes. A total of 12 *S. aureus* isolates exhibited resistance to 8–10 different antibiotics belonging to six different antibiotics classes. Another 12 *S. aureus* isolates showed resistance to 6–9 antibiotics belonging to five antimicrobial classes. In addition, MDR to 4–8 antibiotics belonging to four antimicrobial classes was observed in 12 *S. aureus* isolates. MDR to three and two antibiotic classes was found in five and 10 *S. aureus* isolates, respectively. Taken together, most of the *S. aureus* isolates from animal food products were multidrug-resistant. 

### 3.5. Molecular Characterization of the Staphylococcus aureus Strains

For molecular characterization of the *S. aureus* strains, we first tested the presence of the *coa* gene in the *S. aureus* strains isolated from the animal food products and human pus samples. To this end, we randomly selected nine *S. aureus* strains isolated from minced meat, beef luncheon, Karish cheese, and human swabs samples and amplified the *coa* gene by PCR. The PCR method successfully amplified a 600–1000 nucleotide fragment of the *coa* gene of *S. aureus* strains in all samples (Supplementary Figure S1). Two isolates of minced meat, beef luncheon, and Karish cheese were positive for the *coa* gene. In addition, the *coa* gene was detected in three isolates obtained from human pus samples.

Next, we tested the presence of the *mecA* gene in the *S. aureus* strains isolated from minced meat, beef luncheon, Karish cheese, and human pus samples. For this, we selected 19 *S. aureus* strains that exhibited strong reactions in biochemical tests and virulence factors including coagulase test, DNase-, hemolysis on blood agar, and Biofilm activities. The PCR technique amplified a 583-nucleotide fragment of the *mecA* gene of *S. aureus* strains in 11 isolates (Supplementary Figure S2). The *mecA* gene was detected in two isolates each of minced meat, beef luncheon, and Karish cheese. Five isolates from human pus samples were also positive for the *mecA* gene.

**Figure 2:**
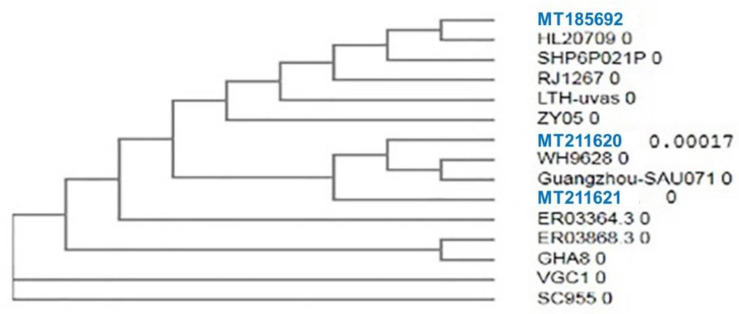
Dendrogram for nucleotide similarity between *Staphylococcus aureus* isolates from minced meat, Karish cheese, and human samples. Sequences (highlighted in blue color) from human samples (MT185692), minced meat (MT 211620), and Karish cheese (MT 211621) were generated in this study.

**Figure 3:**
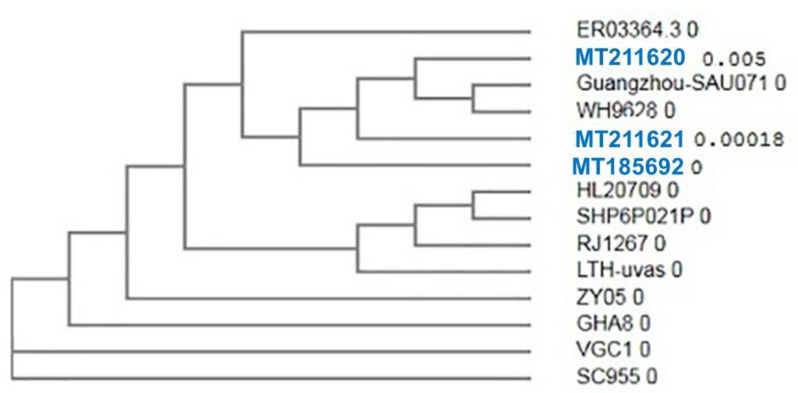
Dendrogram for protein similarity between *Staphylococcus aureus* isolates from minced meat, Karish cheese, and human samples. Sequences (highlighted in blue color) from human samples (MT185692), minced meat (MT 211620), and Karish cheese (MT 211621) were generated in this study.

Then, we tested the genetic diversity of the *mecA* gene in the *S. aureus* strains isolated from animal food products and human samples. We sequenced the partial *mecA* gene of three *S. aureus* strains to analyze the genetic similarities between the *S. aureus* from human and animal food products. The three selected strains of *S. aureus* were obtained from three different sources (human, minced meat, and Karish cheese), were multidrug-resistant strains, and had the same properties as other strains from the same source in terms of morphology and biochemical and virulence activities. The *mecA* partial gene sequences from human, minced meat, and Karish cheese samples were submitted in the GenBank with accession numbers MT185692, MT211620, and MT211621, respectively. The *mecA* gene sequences of our three *S. aureus* strains showed high nucleotide similarity (99.79 to 100%) with *mecA* gene sequences of *S. aureus* reported from different countries. The phylogenetic tree also clustered these isolates with the *S. aureus* isolates obtained from different geographical locations (Figure 2). For example, we detected nearly identical sequences of the *mecA* gene detected in three *S. aureus* strains with the *mecA* genes of *S. aureus* strains detected in human samples from the USA (HL20709; ER03364.3, and ER03868.3), three isolates of pigs and human origin from China (SH6P021P, LTH-uvas, and ZY05), one human specimen from Pakistan (RJ1267), and one human urine sample from Japan (SC955). The protein identity and similarity between our isolates and isolates from other countries are illustrated in the phylogenetic tree (Figure 3). The result showed that nearly identical protein sequences of the *mecA* gene of the study strains have been found in different geographical areas including two isolates from China (Guangzhou-SAu071 and WH9628) obtained from human bile and sputum specimens and with one isolate each from the USA and South Korea obtained from human specimens (ER03364.3 and HL20709). Additionally, the phylogenetic tree clustered our isolates with *S. aureus* isolates from several countries. For example, we observed high amino acid sequence similarities of the *mec*A gene of our study with one isolate from a pig in Pakistan (LTH-uVas) and two isolates from human sputum and blood specimens in China (RJ1267 and ZY05). Furthermore, three human isolates from three different countries showed high genetic similarities with our isolates, including two isolates from Ghana and Taiwan (GHA8 and VGC1) and one from Japan (SC955). 

## 4. Discussion

The *Staphylococcus aureus* is a common bacterial pathogen that can cause severe infectious diseases in livestock animals and humans. *S. aureus* may cause health problems, such as skin and soft tissue infection, septic arthritis, osteomyelitis, pneumonia, and endocarditis [30]. In this study, we found that 35% of the food products and human pus samples carried the coagulase-positive *S. aureus* (CPS). The CPS isolates showed resistance to amoxicillin (91.4%), penicillin (97.1%), cefoxitin (85.7%), cephradine (82.9%), tetracycline (57.2%), and erythromycin (52.8%).

The prevalence of *S. aureus* in minced and beef luncheon was 48% and 84%, respectively. Tang and colleagues [31] reported similar findings, detecting *S. aureus* in 68% of Denmark’s retail meat samples. In addition, a similar study identified *S. aureus* in 51%, 43.35%, and 12.2% of raw meat, quick-frozen meat, and ready to eat meat samples, respectively [32]. A low prevalence of *S. aureus* (16.6%) in meat samples from 60 butcher shops was reported in Egypt [33]. Furthermore, *S. aureus* prevalence of 27.9% was reported in retail meat samples in the USA [34].

In the present study, *S. aureus* was predominant in Karish cheese (76%). This result was consistent with other Egyptian studies that reported *S. aureus* prevalence rates of 93% and 90% from Karish cheese [35,36]. However, a lower *S. aureus* prevalence of 30% and 24% in Karish cheese in Egypt was also recorded [1,37]. The prevalence rate of *S. aureus* in human pus samples was 38% in the present study. This is in line with a previous study [38], which isolated *S. aureus* in 11.3% of patients from sputum specimens. The wide range in the reported prevalence of *S. aureus* in meat, milk products, and human samples among local and global studies may be attributed to differences in the hygienic measures implemented during food processing and manufacturing in the region of the study. Additionally, the handling procedure and hygienic condition surrounding the product manufacture can affect the degree and percentage of microbial contamination; for example, in Karish cheese, which is a type of handmade cheese (made of raw milk or laban rayeb with salt, produced without heat treatment) in popular use and is manufactured in most areas in Egypt. Furthermore, minced meat and beef luncheon are also manufactured from frozen or raw meat and the processing involves no or low heat treatment. 

Based on the biochemical and enzymatic activity of the obtained coagulase-positive *Staphylococci* (CPS) isolates, hemolytic and DNase activities were detected in all sample types. Lecithinase activity was detected in minced meat, beef luncheon, and human samples; however, no Karish cheese isolates exhibited lecithinase activity. Similarly, Ali and colleagues [39] recorded DNase activity in 86.9% of the CPS. Furthermore, DNase activity and coagulase activity was observed among 60% and 87.5% of the CPS, respectively, as reported previously [37,40].

The molecular identification of the *coa* gene in *S. aureus* strains was performed through amplification of the *coa* gene at (600–1000) bp. The use of the *coa* gene to detect *S. aureus* strains from milk origin was previously reported in two studies [37,41] which amplified the *coa* gene from mastitic milk and dairy products at 630 and 750 bp. Moreover, Javid and colleagues [42] detected the *coa* gene at 514 bp, 595 bp, 757 bp, and 802 bp.

In recent years, the widespread use of antibiotics to treat bacterial infections has led to the emergence of multidrug-resistant (MDR) bacteria, which constitute a great hazard to public health. For example, *S. aureus* could adapt to adverse environmental conditions, leading to the emergence of many strains resistant to different antibiotic classes [43]. In the current study, the phenotypic testing of 70 *S. aureus* isolates from meat and milk products, and humans revealed a high prevalence of resistance against penicillin (97.1%), amoxicillin (91.4%), cefoxitin (85.7%), cephradine (82.9%), tetracycline (57.2%), and erythromycin (52.8%). Similar findings were obtained by Mohammed and Hafez [33] in Egypt, who detected high resistance against chloramphenicol and ampicillin (100%), streptomycin (66.6%), gentamicin and trimethoprim-sulphamethoxazole (33.3%), and erythromycin (16.6%). In a recent study in Egypt, *S. aureus* demonstrated extreme resistance to cefoxitin, penicillin, cephradine, tetracycline, trimethoprim-sulphamethoxazole, and norfloxacin in samples from raw milk and ice cream [4]. In China, Wu and colleagues [32] also recorded high resistance of *S. aureus* isolated from retail meat and meat products against penicillin (84.6%), ampicillin (85.4%), erythromycin (52.7%), tetracycline (49.3%), kanamycin (45.3%), telithromycin (30.1%), clindamycin (29.6%), streptomycin (21.1%), norfloxacin (20.4%), gentamicin (19.4%), fusidic acid (18.4%), and ciprofloxacin (16.9%). Most of the published studies supported our findings that *S. aureus* has become a notorious pathogen due to its high resistance against many antimicrobial agents. Therefore, more studies are required to overcome this problem in veterinary or human practices.

Methicillin-resistant *S. aureus* (MRSA) is a pathogen causing human and livestock animal infections. MRSA contaminates many types of food and dairy products, which cause foodborne poisoning [44] and the detection of MRSA in animal-derived foods is necessary for the monitoring and improvement of hygiene measures in food practices to diminish the potential for bacterial contamination [44]. The results in Figure 1 demonstrated a high prevalence and emergence of MRSA strains from meat, milk products, and human samples, constituting a major public health hazard. These are consistent with several other studies [45,46]. Interestingly, the existence of MRSA in the environment can generally allow the dissemination and spread of MRSA in animal products [47].

The *mecA* gene is known to encode penicillin-binding protein 2a (PBP2a), which is responsible for resistance against beta-lactam [16]. Modern molecular techniques, such as PCR, are used for *mecA* gene detection and is recognized as a specific method for the detection of MRSA strains among staphylococcal species [48]. In this study, the *mecA* gene was successfully amplified in 11 out of 19 tested isolates (57.89%) at 583 bp. A comparative study in Nigeria reported that the *mecA* gene was prevalent in 38% of *S. aureus* isolates from human clinical specimens [49]. In Egypt, 8 out of 19 (42.1%) food origin *S. aureus* isolates exhibited phenotypic resistance to oxacillin and carried the *mecA* gene [50]. A similar finding was reported by Awad and colleagues [51], who showed that 50% of *S. aureus* isolates of mastitis origin harbored the specific *mecA* gene and expressed phenotypic resistance to multiple antimicrobials groups. In addition, the existence of the *mecA*-positive MRSA in bovine milk strains was described earlier in the USA [52] as well as in Denmark [31].

The sequences analysis of the *mecA* gene originated from three different sources in this study (minced meat, Karish cheese, and human pus) revealed high genetic similarities (99.79 to 100%) with many isolates from different nations and localities both at nucleotides and protein level. This result was consistent with Malik and colleagues [53], who reported that identical homologs of the *mecA* gene of MRSA strains originated from human, cat, and dog, which is attributed to the close contact between humans and these animals. Moreover, these resistance genes are disseminated between animals and humans due to the normal inhabitance of *Staphylococci* in the skin and respiratory mucosa, which could easily be dispersed by aerosols from sneezing and coughing, skin-to-skin contact, and saliva [53]. In a Brazilian study, phylogenetic analysis of the *mecA* gene nucleotide sequences from 210 skin and ears rodent samples and 60 equine nasal samples separated them into two distinct groups in the phylogenic tree, one of which belongs to the bovine clones and the other related to the equine and human strains [54]. Interestingly, the authors declared that the *mecA* from equine *S. sciuri* and *S. lentus* was highly homologous to the *mecA* from human *S. aureus*, whereas a clear diversity from bovine *S. sciuri* was noticed, suggesting that the evolution of a resistance gene is more likely related to the host environment rather than the bacterial species. However, the presence of another mobile genetic element called staphylococcal chromosome cassette *mec* (SCC*mec*) in MRSA strains of human origin suggests that the *mecA* gene could be a shared gene among different staphylococcal species [55]. Regarding the dissemination of the *mecA* gene in both human and food samples, Velasco and colleagues [56] demonstrated the similarity between 108 clinical MRSA isolates and 133 *S. aureus* isolates from animal origin and clarified the high genetic similarity between the human and meat clones. The genetic similarity between human and meat strains suggests contamination of raw meat during handling and the potential risk of *S. aureus* spread through the food chain. High genetic similarity among *S. aureus* strains from different sources such as meat-producing animals and retail meat could be attributed to meat contamination during slaughtering [57]. However, Sung and colleagues [58] showed that clones of *S. aureus* among animals are closely related to human clones. This could be due to the adaptive behavior mechanism of *S*. *aureus* isolates [59]. In Iraq, Neamah and colleagues [60] found a high genetic linkage between *S. aureus* from humans and cattle and a major genetic link between livestock animals and human isolates.

## 5. Conclusions

The *S. aureus* was prevalent among meat, milk products, and human samples. Our results highlighted the predominance of MRSA strains with resistance to multiple antimicrobial agents in animal food products. In addition, the genetic analysis of the *mecA* gene revealed high nucleotide and amino acid similarities between *S. aureus* strains from human samples and food products. Additionally, the study detected the presence of the *mecA* gene in *S. aureus* isolates from humans and animal food products (minced meat and Karish cheese) in the study area in Egypt and its possible threat to public health. Therefore, frequent monitoring of *S. aureus* to identify patterns of antimicrobial susceptibility is needed to elucidate MRSA transmission routes through the animal food chain. In addition, it is necessary to follow up the hygienic measures to prevent or minimize the contamination of food products, particularly that of handmade products in the study area, which constitute a high risk on public health.

## Figures and Tables

**Table 2:** Prevalence of coagulase-positive *Staphylococci* (CPS) isolated from food and human samples.

Human Swabs (*n* = 50)		Karish Cheese (*n* = 50)		Beef Luncheon (*n* = 50)		Minced Meat (*n* = 50)	
CPS		CPS		CPS		CPS	
%	No.	%	No.	%	No.	%	No.
38	19	76	38	84	42	48	24

% estimated according to the number of each sample (*n* = 50).

**Table 3:** Antimicrobial resistance of the CPS isolates.

Antibiotics	Antimicrobial Classes	Resistant		Intermediate		Sensitive	
		No.	%	No.	%	No.	%
Penicillin (P) 1 IU	β-lactams	68	97.1	0	0	2	2.9
Amoxicillin (AX) 25 µg	β-lactams	64	91.4	0	0	6	8.6
Cefoxitin (FOX) 30 µg	β-lactams	60	85.7	0	0	10	14.3
Vancomycin (VA) 30 µg	Glycopeptides	10	14.3	1	1.4	59	84.3
Gentamicin (CN) 10 µg	Aminoglycosides	30	42.8	9	12.9	31	44.3
Erythromycin (E) 15 µg	Macrolides	37	52.8	30	42.9	3	4.3
Tetracycline (TE) 30 µg	Tetracyclines	40	57.2	8	11.4	22	31.4
Ciprofloxacin (CIP) 5 µg	Fluoroquinolones	23	32.9	17	24.3	30	42.8
Norfloxacin (NOR) 10 µg	Fluoroquinolones	17	24.3	14	20	39	55.7
Trimethoprim-Sulfamethoxazole (SXT) 1.25/23.75 µg	Sulfonamides	29	41.4	6	8.6	35	50
Chloramphenicol (C) 30 µg	Chloramphenicol	18	25.7	3	4.3	49	70
Cephradine (CE) 30 µg	β-lactams	58	82.9	0	0	12	17.1

% estimated according to the number of tested isolates for antibiotic sensitivity test (*n* = 70).

## Data Availability

All authors agree that the data presented in this study are openly available through mdpi publisher platform or others without any restriction.

## Supplementary Materials

The following are available online at https://www.mdpi.com/2079-6382/10/1/75/s1, Table S1: Multi-drug resistant S. aureus strains from food and human samples. Figure S1. Amplification of the coa gene of Staphylococcus aureus at 600–1000 bp: Lane M: 100 bp DNA ladder. Pos.: Positive control. Neg.: Negative control. Samples 1 and 2 from minced meat, 3 and 4 from beef luncheon, 5 and 6 from Karish cheese, and 7, 8, and 9 from human were recorded as positive samples for the coa gene. Supplementary Figure S2. Amplification of the 583 bp fragment of the mecA gene of Staphylococcus aureus; Lane M: 100 bp DNA ladder. Pos.: Positive control. Neg.: Negative control; Lane: Samples 3 and 5 from minced meat; 7 and 8 from beef luncheon; 9 and 12 from Karish cheese; 13, 14, 15, 18, and 19 from human samples were recorded as positive samples for the *mecA* gene.
