# Supplementary material for: Prevalence and Characterization of Coagulase Positive Staphylococci from Food Products and Human Specimens in Egypt
Source: Antibiotics (Basel). 2021 Jan 14;10(1):75. doi: 10.3390/antibiotics10010075 (PMC7829985; doi:10.3390/antibiotics10010075)
Supplement: Supplementary file 1 [file antibiotics-10-00075-s001.pdf]

**Supplementary materials**

**Supplementary Table S1.** Multi-drug resistant *S. aureus* strains from food and human samples.

| Resistant isolates | | Resistance profile | Antibiotics classes | No. of resistance antimicrobial classes |
|---|---|---|---|---|
| Nº | % | | | |
| 2 | 4.17 | P, AX, VA, FOX, CE, CN, E, TE, CIP, NOR, SXT, C | β-lactams, Aminoglycosides, Glycopeptides, Tetracyclines, Fluoroquinolones, Sulfonamides, Chloramphenicol | **8** |
| 2 | 4.17 | P, AX, FOX, CE, CN, E, TE, NOR, SXT, C | β-lactams, Aminoglycosides, Macrolides, Tetracyclines, Fluoroquinolones, Sulfonamides, Chloramphenicol | **7** |
| 4 | 8.33 | P, AX, FOX, CE, CN, TE, CIP, NOR, SXT, C | β-lactams, Aminoglycosides, Tetracyclines, Fluoroquinolones, Sulfonamides, Chloramphenicol | **6** |
| 16 | 33.33 | P, AX, FOX, CE, CN, E, TE, SXT | β-lactams, Aminoglycosides, Macrolides, Tetracyclines, Sulfonamides | **5** |
| 11 | 17.7 | P, AX, VA, FOX, CE, CN, TE | β-lactams, Glycopeptides, Aminoglycosides, Tetracyclines | **4** |
| 13 | 27.08 | P, AX, FOX, CE, CN, CIP | β-lactams, Aminoglycosides, Fluoroquinolones | **3** |

% estimated according to the number of tested isolates for antibiotic sensitivity test (n=70)

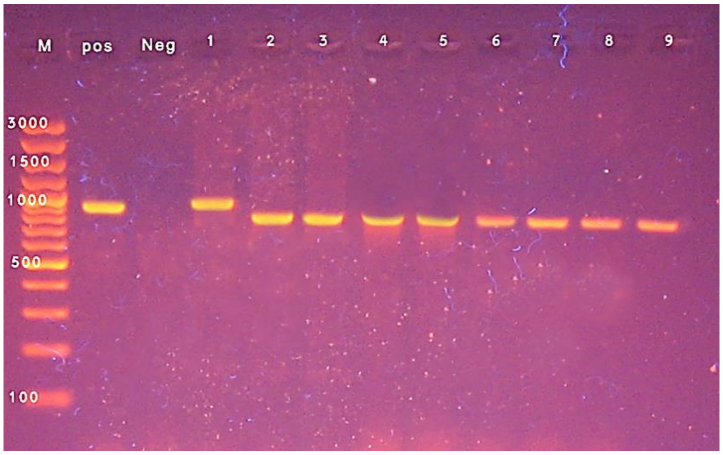


**Supplementary Figure S1.** Figure S1. Amplification of the *coa* gene of *Staphylococcus aureus* at 600–1000 bp: Lane M: 100 bp DNA ladder. Pos.: Positive control. Neg.: Negative control. Samples 1 and 2 from minced meat, 3 and 4 from beef luncheon, 5 and 6 from Karish cheese, and 7, 8, and 9 from human were recorded as positive samples for the *coa* gene.

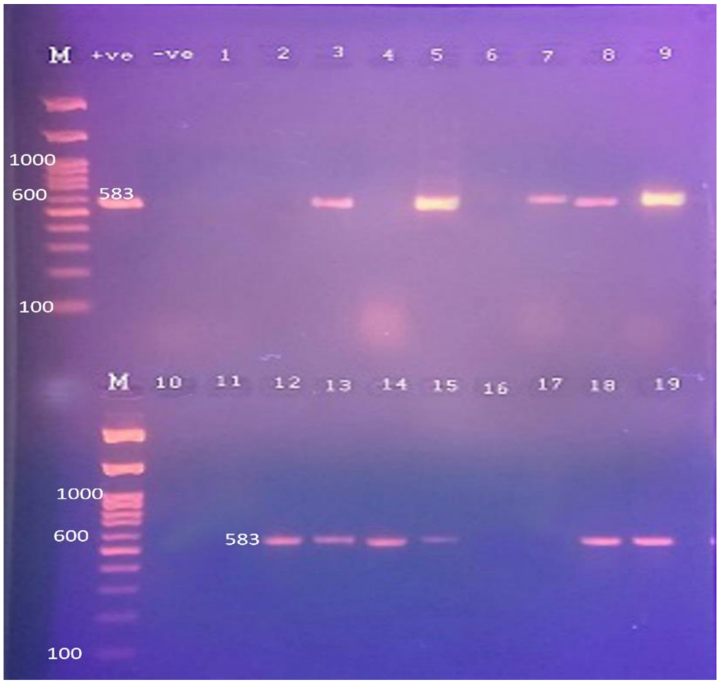


**Supplementary Figure S2.** Amplification of the 583 bp fragment of the *mecA* gene of *Staphylococcus aureus*; Lane M: 100 bp DNA ladder. Pos.: Positive control. Neg.: Negative control; Lane: Samples 3 and 5 from minced meat; 7 and 8 from beef luncheon; 9 and 12 from Karish cheese; 13, 14, 15, 18, and 19 from human samples were recorded as positive samples for the *mecA* gene.
